# Ethnobotanical study of cowpea (*Vigna unguiculata* (L.) Walp.) in Senegal

**DOI:** 10.1186/s13002-022-00506-y

**Published:** 2022-02-05

**Authors:** Awa Sarr, Amy Bodian, Mame Codou Gueye, Badara Gueye, Ghislain Kanfany, Cyril Diatta, Lardia Ali Bougma, Elisabeth A. M. C. Diop, Ndiaga Cissé, Diaga Diouf, Christian Leclerc

**Affiliations:** 1 Centre d’Etude Régional pour l’Amélioration de l’Adaptation à la Sécheresse (CERAAS)/Institut Sénégalais de Recherches Agricoles (ISRA), BP 3320, Thiès, Sénégal; 2 Laboratoire Campus de Biotechnologies Végétales, Département de Biologie Végétale, Faculté des Sciences et Techniques, Université Cheikh Anta Diop (UCAD), BP 5005, Code Postal 10700 Dakar Fann, Dakar Sénégal; 3 International Institute of Tropical Agriculture (IITA), (BP) Ibadan, Nigeria; 4 Centre National de Recherches Agronomiques (CNRA)/Institut Sénégalais de Recherches Agricoles (ISRA), BP 211, Bambey, Sénégal; 5 Département de Biologie et Physiologie Végétales, Université Joseph KI-ZERBO, BP 7021, Ouagadougou 03, Burkina Faso; 6 CIRAD, UMR AGAP Institut, 34398 Montpellier, France; 7 UMR AGAP Institut, Université de Montpellier, 34398 Montpellier, France

**Keywords:** Cowpea, Ethnobotanical, Local names, Farming systems, Senegal

## Abstract

Cowpea (*Vigna unguiculata*) plays a key role in family farming systems in Senegal. It makes an essential contribution to economic, nutritional and food security. Although it is crucial, little is known about how farmers classify the diversity of local varieties or about the social practices associated with them. The aim of this study is to characterize the farming practices associated with growing cowpea in Senegal. Surveys were conducted involving 335 rural farmers living in 37 villages, spread across seven regions that produce cowpea. An average of ten farmers were randomly selected in each village. The results reveal that cowpea is a key feature of cropping systems in the studied area. Our findings highlight the high diversity of local cowpea varieties with 59 local names inventoried. In 75% of cases, the name refers to the seed’s morphology or color. Cowpea production is more diverse in Diourbel and Louga and less diverse in the south. More than half the farmers (57%) acquired their cowpea seeds (early, semi-early and late maturity varieties) outside their village, either from markets, seed suppliers or NGOs. This new understanding of farmers’ expertize in the management of cowpea and its local variability will help to valorize local diversity in breeding programs.

**Supplementary Information:**

The online version contains supplementary material available at 10.1186/s13002-022-00506-y.

## Introduction

Cowpea (*Vigna unguiculata* (L.) Walp.) is one of the most important leguminous plant grown in tropical savannah zones in Africa [1]. Its cultivation makes a major contribution to food security for people living in the most marginal areas. Its seeds are rich in lysine and tryptophan, which are a valuable source of plant protein [2]. In addition, cowpea is an essential source of vitamins and minerals, which help to prevent birth defects [3, 4]. Its capacity to fix atmospheric nitrogen improves soil fertility and helps to reduce the need of chemical fertilizer [5, 6]. Cowpea is one of the legumes most often grown in association with cereals in rural areas. Several studies conducted in sub-Saharan Africa have shown that pulses, like cowpea, have a positive effect on cereal yield [7–10]. Young leaves and immature pods are eaten as a vegetable, and the haulms are used as livestock fodder [11, 12]. Cowpea cultivation generates income through the sale of green pods and fresh seeds during the lean season and fodder, especially during the dry season when it is sold at twice the price. Formerly considered as a subsistence crop, it is now grown as cash crop and has a major socio-economic impact on Sahelian countries as in Senegal where the crop is growing on 290,677 hectares with annual production over 180 000 tonnes in 2019 [13]. Usually, women make the cowpea harvest, sale and processing (couscous, thiakry, cake, coffee, etc.).

Despite the fact that its social and economic value has been demonstrated, our knowledge of the diversity of the varietal forms grown in family farming systems remains limited. Historically, local early flowering cowpea varieties were introduced from Nigeria for floodplain cropping in the Senegal River Valley, in the north of the country. In contrast, some late flowering varieties were introduced from Mali and grown in association with millet in more humid regions in Senegal [14]. These varieties spread to the rest of the country as a result of trade and migration. Today, cowpea is mainly produced in the center and central north of the country [15].

Despite the key role of cowpea in Senegalese farming systems, little is known about the local management of cowpea. The ethnobotanical classification of cowpea diversity is essential for improving the conservation (in situ or ex situ) and valorization of this legume. It is particularly relevant for breeding programs, which require the availability of a wide genetic diversity [16]. In this respect, local cowpea varieties constitute a heritage of major importance. The surveys and/or collections that allowed us to identify cowpea varieties in the past focused on a limited number of regions. Cowpea collections were established between 1953 and 2003 in Senegal [14, 17, 18]. However, these accessions have been partially lost rising the need to establish a new cowpea collection.

Based on new collections and specific more exhaustive surveys, this study aims to characterize the farming practices associated with growing cowpea in Senegal for the first time. In particular, it aims to: (i) identify the role that cowpea has in the cropping system, by describing the range of species that it is associated with; (ii) survey and characterize its diversity based on the local nomenclature and the date to reach maturity and (iii) identify the farmers’ seed supply.

## Materials and methods

### Study areas and sampling strategy

**Fig. 1:**
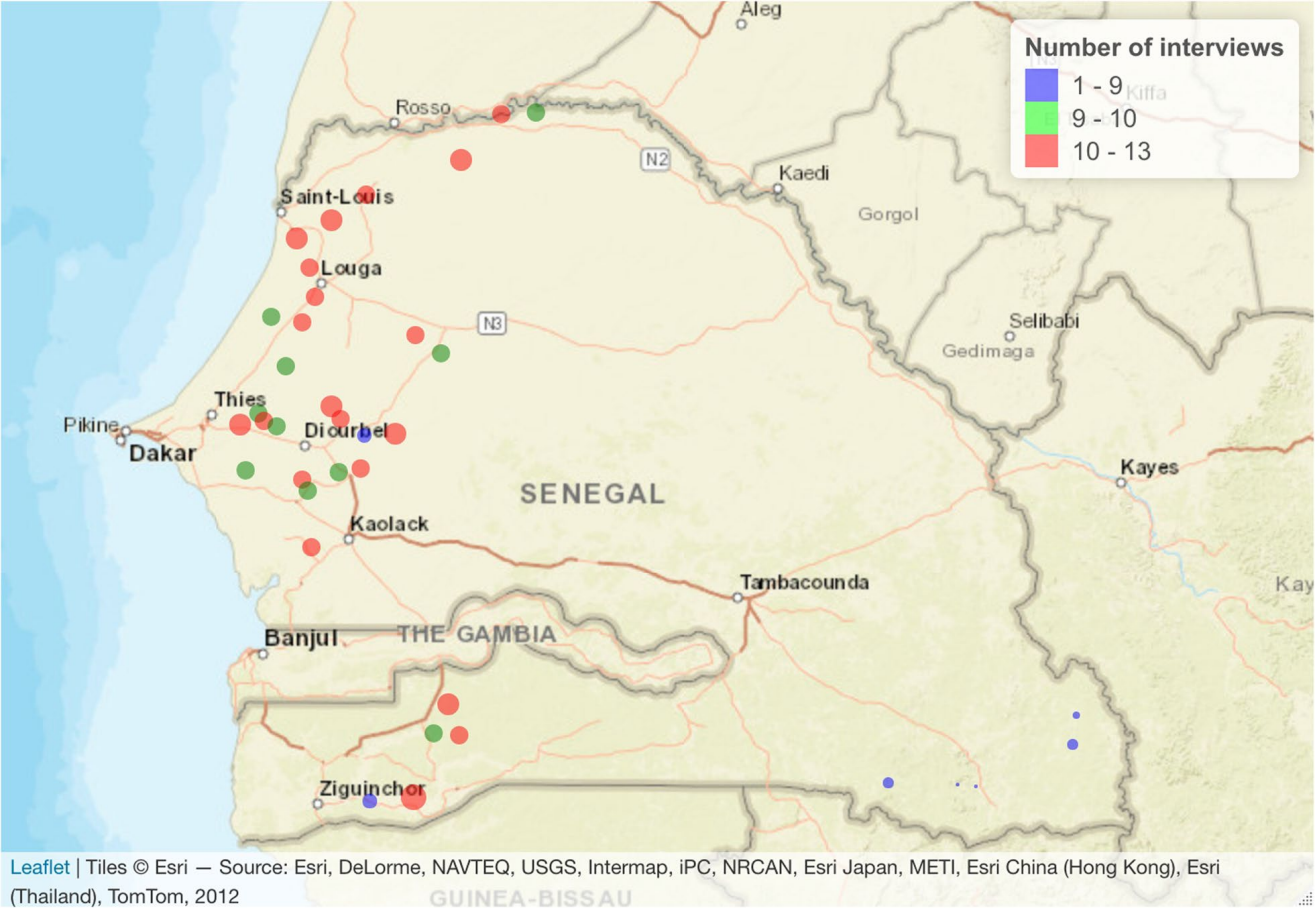
Location of villages surveyed. The size of the dots is proportional to the number of people surveyed

The surveys were conducted between September 2015 and March 2016 in the main cowpea producing regions in Senegal (Louga, Thiès, Fatick, Diourbel, Sédhiou and Saint-Louis). The Kédougou region was also surveyed in order to identify the characteristics of the cowpea varieties grown in this area. The villages surveyed were chosen in consultation with agents from the services of Regional Rural Development Division to facilitate access to villages that grow cowpea. To optimize the coverage of the main cowpea producing zones, three departments were visited in each region (Fig. 1). The sampling strategy also aimed to provide the best representation of the diversity of ethnic groups that grow cowpea, based on the assumption that farming practices may vary from one group to another [19]. Thus, we selected average-sized villages in different communes, located at least 15 km apart and 10 km from the national road and the market. The survey was organized in 37 villages, from four to six villages per region (Additional file 1).

### Surveys on cropping diversity

In each village surveyed, participatory research methods and tools were applied to find out about cowpea management and the varietal diversity used by farmers during the 2015 rainy season [20]. The floodplain cultivation system in the Saint-Louis Region was also considered (October 2015–February 2016). The survey was conducted with the help of informal and semi-structured interviews, in addition to focus group discussions. The informal interviews were initially conducted with the village chief to find out about the site’s socio-cultural and demographic characteristics. The semi-structured interviews were conducted based on a questionnaire. This type of interview involves a discussion between the surveyor and the interviewee, which allows for reminders and interactions [21].

The semi-structured interviews made it possible to identify the range of species associated with cowpea (Additional file 2), describe the varietal diversity of cowpea using the local names and determine seed origin and the cowpea accession cycles (Additional file 3) (accessions were collected at the same time). A free listing method was used [22] to identify the diversity of species grown with cowpea, as well as the diversity of specific cowpea morphotypes or local varieties. Here, the term “variety” corresponds to local names to designate local morphotypes. These morphotypes that farmers considered as landraces are accessions and not taxonomic varieties. Their importance is evaluated in relation with their frequency. Free listing is a technique that is widely used in ethnobotanical studies. It involves asking farmers to list all the known varieties for a given species [23]. This technique is used to explore and test their knowledge regarding cowpea classification. It was used to classify the species and varieties of cowpea grown by farmers during the rainy season. As Henley Henley (1969) and Borgatti (1999) pointed out, “the order in which elements are listed by individual respondents is not arbitrary.” A first list of species (or varieties) is proposed by farmers with no hesitation. After a pause, a second complementary list is proposed, following by a third one and so on. The aim is to interpret these different series, by taking into account the order in which the species (or varieties) are listed by each interviewee. The hypothesis is that the most important species (or varieties) tend to be mentioned first.

The focus groups, which brought together about ten farmers in the public square or at the village chief’s home, made it possible to check whether the full range of crop diversity in the village had been identified during the individual interviews.

The spelling for the local names of the cowpea varieties was harmonized, and the synonyms were identified to ensure that only one term of reference was kept and translated into English.

### Collecting the cowpea accessions

After each individual interview, all the cowpea accessions grown by the farmer were collected. An accession corresponds to the name of a variety grown by a farmer. In fact, after recording the names of varieties grown by a farmer, a visit to the field and/or storage area was conducted to collect samples. Ideally, pod samples were taken from the field. Otherwise, seeds were sampled in the granary.

The accessions collected were put in envelopes, labelled and kept before being transported to the “Centre d'Etudes Régional pour l'Amélioration à l'Adaptation à la Sécheresse” (CERAAS) in Senegal for conservation.

The villages’ geographic coordinates were recorded on a tablet with the aid of the software Sygic: GPS Navigation 17.3.27 Android.

### Data analysis

The age, ethnic group and profession were used to characterize the farmers interviewed. The frequency, the average salience and Smith’s index for each species and variety were calculated with the R AnthroTools package [24]. The frequency with which an element was cited (species and varieties) reflects its importance and its perceptual distinctive character. Salience is determined by order of citation; an element is more important when cited at the beginning of the list [25]. Smith’s index is a weighted average of the reverse order for each element. A correspondence analysis was conducted between these species and the different regions of the study.

The number of cowpea morphotypes that farmers identified and named was used to estimate the varietal richness [26]. To further understand the cowpea cultivation, we described the practices associated with each morphotype that was identified, in particular, how seeds were obtained (place of origin of seeds) and the cropping method (single or multiple cropping). A more detailed analysis of the local names used by farmers made it possible to describe the naming process and identify the main categories of cowpea names.

The farmers’ responses regarding the sowing and harvest dates allowed to propose a classification system according to the phenology of the cowpea varieties. The association between the variety types and the regions was checked using a Chi-square test. The maps showing the village locations and the spatial distribution of the accessions were compiled using the software R (version 3.6.0 for Windows). The software packages *Stats* and *FactoMinR* were used for exploratory statistical analyses and to test the hypothesis.

## Results

### Socio-cultural and demographic characteristics of the interviewees

The panel of interviewees comprised 156 women and 179 men, for a total of 335 people. In the different regions, on average, about ten farmers were randomly selected per village—except in the Kédougou Region, where it was only possible to interview four farmers per village (Table 1).

**Table 1:** Number of villages and farmers surveyed per region

Regions	Number of villages	Average number of farmers	Total number of farmers
Diourbel	6	9.8	59
Fatick	5	9.6	48
Kédougou	5	3.6	18
Louga	6	9.7	58
Saint-Louis	6	10.3	62
Sédhiou	5	10.4	52
Thiès	4	9.5	38
Total	37	9	335

The average age of interviewees was 48 years, with no significant difference between the regions, except in the Sédhiou region, where the average age was lower than elsewhere (37 years). Among those interviewed, 50.8% spoke Wolof, which is the language mainly spoken in the regions of Thiès, Louga, Diourbel and Saint-Louis. The Serer, which represented 17.9% of the interviewees, are found in the Fatick, Thiès and Diourbel regions. Lastly, the Toucouleur (10.5%) and Moors (3.3%) occupy the Louga and Saint-Louis regions, while the Mandinka, Jola, Bainuk, Bedick and Manjak live in the Fatick, Kédougou and Sédhiou regions (Table 2).

**Table 2:** Characteristics of the farmers interviewed in each region

		Regions							Total	%
Variables	Modalities	Th	Lg	Dl	Fk	Sd	Kg	SL		
Age	< 25	2	1	0	0	2	1	3	9	2.68
	25–50	20	30	19	17	43	8	25	162	48.36
	50–75	15	26	36	28	7	9	32	153	45.67
	≥ 75	1	1	4	2	0	0	2	10	2.98
	NA	0	0	0	1	0	0	0	1	0.3
	Total	38	58	59	48	52	18	62	335	100
	Wolof	22	35	40	9	31	0	33	170	50,75
Ethnic group	Mandinka	0	0	0	3	7	10	0	20	5.97
	Moor	0	3	0	0	0	0	8	11	3.28
	Fulani	0	10	4	2	0	6	1	23	6.86
	Serer	15	1	13	31	0	0	0	60	17.91
	Toucouleur	1	9	2	3	0	0	20	35	10.45
	Other	0	0	0	0	14	2	0	16	4.77
	Total	38	58	59	48	52	18	62	335	100

*Th* Thiès; *Lg* Louga; *Dl* Diourbel; *Fk* Fatick; *Sd* Sédhiou; *Kg* Kédougou; *SL* Saint-Louis; *NA* data not provided

### Cowpea cropping systems

Twenty-four (24) different species grown with cowpea were identified in the seven regions which were studied. The most frequently cited species grown with cowpea were groundnut and millet, which on average are grown, respectively, by 85% and 71% of the farmers interviewed (Table 3). However, the proportion of farmers that grow groundnut or millet varies depending on the region. While 98% of farmers grow groundnut in Diourbel, the figure is only 43% in Saint-Louis. This variation is also observed for millet, which is common in Diourbel and Louga, but more unusual in Saint-Louis. Other crops are far less common than these two species, such as guinea sorrel, maize, watermelon, rice and sorghum. Their distribution also varies depending on the region. The least common species grown with cowpea (only 0.3% of farmers interviewed) are calabash (Kédougou), cucumber and melon (Saint-Louis), turnip (Thiès) and Bambara groundnut (Diourbel).

**Table 3:** Different species grown and their percentage in each region

Species common name	Species Latin name	Diourbel	Fatick	Kédougou	Louga	Saint-Louis	Sédhiou	Thiès	Total
Bambara nut	*Vigna subterranea* (L.) Verdc	1.7	0	0	0	0	0	0	0.3
Calabash	*Lagenaria siceraria* (Molina) Standl	0	0	5.6	0	0	0	0	0.3
Cassava	*Manihot esculenta* Crantz	0	0	27.8	0	8.1	13.5	0	5.1
Chili	*Capsicum annuum* (L.)	0	0	0	1.7	4.8	3.8	0	1.8
Cotton	*Gossypium *(L.)	0	0	33.3	0	0	0	0	1.8
Cowpea	*Vigna unguiculata* (L.) Walp.	100	95.8	94.4	100	98.4	100	100	98.8
Cucumber	*Cucumis sativus* (L.)	0	0	0	0	1.6	0	0	0.3
Eggplant	*Solanum melongena* (L.)	1.7	0	33.3	0	3.2	0	5.3	3.3
Fonio	*Digitaria exilis* (Kippist) Stapf	0	0	33.3	0	0	0	0	1.8
Groundnut	*Arachis hypogaea* (L.)	98.3	95.8	100	94.8	43.5	84.6	94.7	84.8
Maize	*Zea mays* (L.)	13.6	29.2	94.4	5.2	22.6	55.8	2.6	25.7
Melon	*Cucumis melo* (L.)	0	0	0	0	1.6	0	0	0.3
Okra	*Abelmoschus esculentus* (L.) Moench	5.1	0	66.7	0	6.5	0	2.6	6
Onion	*Allium cepa* (L.)	0	0	5.6	0	22.6	0	5.3	5.1
Pearl millet	*Pennisetum glaucum* (L.) R. Br.	100	79.2	44.4	87.9	24.2	69.2	84.2	71.3
Pumpkin	*Cucurbita* (L.)	5.1	0	33.3	0	6.5	0	0	3.9
Red sorrel	*Hibiscus sabdariffa* (L.)	47.5	35.4	38.9	32.8	35.5	0	21.1	30.1
Rice	*Oryza glaberrima* Steud	0	16.7	77.8	0	12.9	23.1	0	12.5
Sesame	*Sesamum indicum* (L.)	0	0	0	0	0	28.8	0	4.5
Sorghum	*Sorghum bicolor* (L.) Moench	18.6	12.5	55.6	17.2	1.6	0	2.6	11.6
Sweet potato	*Ipomoea batatas* (L.) Lam	0	0	0	0	12.9	7.7	0	3.6
Tomato	*Solanum lycopersicum* (L.)	0	0	5.6	0	6.5	0	0	1.5
Turnip	*Brassica rapa* (L.)	0	0	0	0	0	0	2.6	0.3
Watermelon	*Citrullus lanatus* Thunb	3.4	8.3	0	20.7	62.9	5.8	10.5	19.1
Other		0	0	0	0	3.2	0	0	0.6

**Fig. 2:**
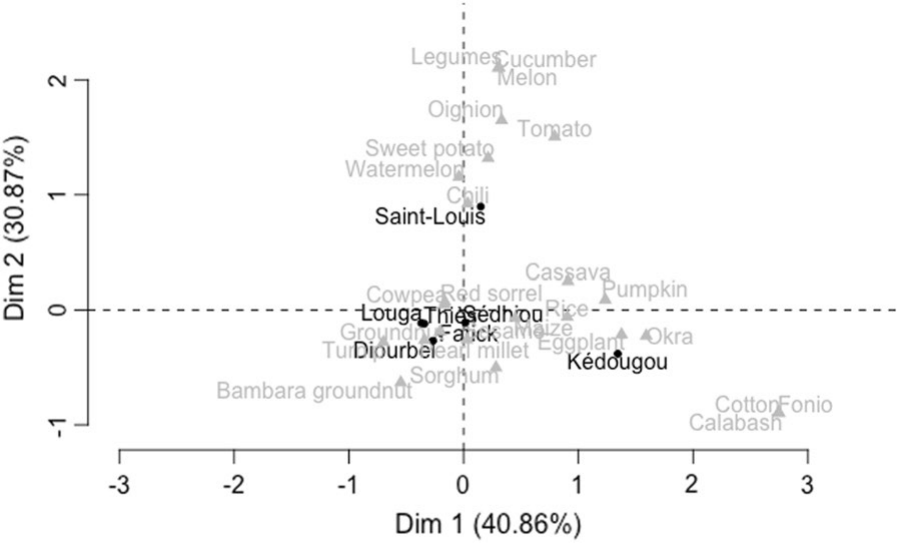
Factor map of correspondence analysis. Association of species as a function of regions surveyed

The correspondence analysis shows that the regions of Thiès, Sédhiou, Louga, Fatick and Diourbel have similar cropping profiles: red sorrel, sesame and sorghum, in addition to cowpea, groundnut and pearl millet. The Saint-Louis region differs, with watermelon (grown by 62.9% of interviewees) and onion (22.6%), melon, cucumber and tomato, whereas the Kédougou region is characterized by fonio, pearl millet and cotton, rarely grown elsewhere (Fig. 2). The three main species (cowpea, groundnut and pearl millet) are not randomly distributed between the regions. However, the disparity only concerns Saint-Louis, where quite a high proportion of farmers grow cowpea compared to what was expected randomly (residual > 3), although this proportion is low for millet (residual > 2.5, X-squared = 26.949, df = 12, *p*-value = 0.008).

**Fig. 3:**
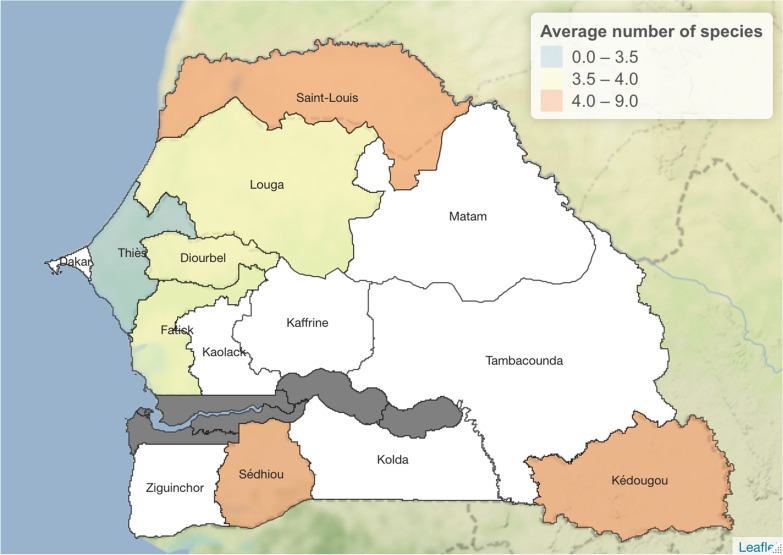
Average number of species grown per region

The number of species cultivated is structured according to the regions (Fig. 3) and varies between three and nine species per farmer in the Thiès and Kédougou regions, respectively. On average, more than four species are grown per farmer in the regions of Kédougou, Saint-Louis and Sédhiou, whereas the number is between 3.5 and 4 per farmer in the regions of Louga, Diourbel and Fatick. Thiès is the region where the average number of species per farmer is the lowest (equal to 3.5) (Table 4).

**Table 4:** Number of species grown per region and the average ratio per farmer

Regions	Total number of species citations	Number of farmers	Average number of species/farmer
Diourbel	233	59	3.95
Fatick	182	48	3.79
Kédougou	155	18	8.61
Louga	210	58	3.62
Saint-Louis	254	62	4.10
Sédhiou	208	52	4.00
Thiès	132	38	3.47

**Fig. 4:**
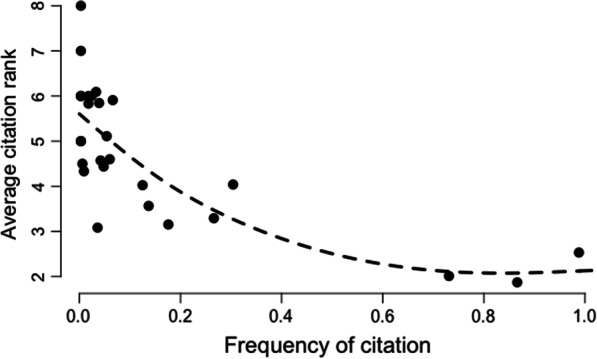
Average citation rank as a function of frequency of citation

Using the free listing method, we established the frequency, Smith’s S index and average salience for each of the species. Groundnut and millet were the species cited the most often with cowpea. The Smith’s index was higher for these three species, with 0.682 for groundnut, 0.612 for cowpea and 0.559 for millet (Table 5). Cowpea is the third most important species in the zones visited, with a citation rank of 2.5, after groundnut (1.9) and millet (2). As expected [22], the citation rank obtained in the species free list is correlated with species frequency in a nonlinear way (Fig. 4).

**Table 5:** Frequency, mean citation rank, Smith’s index, Sutrop index and B.score for species grown with cowpea

Cited items	Latin name	N	Frequency	Mean rank	Smith’s index	Sutrop index	B. score
Cowpea	*Vigna unguiculata* (L.) Walp.	331	0.988	2.532	0.6122	0.3902	0.7209
Bambara groundnut	*Arachis hypogaea* (L.)	290	0.866	1.872	0.6825	0.4624	0.7384
Pearl millet	*Pennisetum glaucum* (L.) R. Br.	245	0.731	2.012	0.5578	0.3635	0.6122
Red sorrel	*Hibiscus sabdariffa* (L.)	102	0.304	4.039	0.1087	0.0754	0.1743
Maize	*Zea mays* (L.)	89	0.266	3.292	0.1487	0.0807	0.1902
Watermelon	*Citrullus lanatus* Thunb	59	0.176	3.153	0.0893	0.0559	0.1148
Rice	*Oryza glaberrima* Steud	46	0.137	3.565	0.0788	0.0385	0.0998
Sorghum	*Sorghum bicolor* (L.) Moench	42	0.125	4.024	0.0557	0.0312	0.0804
Okra	*Abelmoschus esculentus* (L.) Moench	22	0.066	5.909	0.0223	0.0111	0.0388
Onion	*Allium cepa* (L.)	20	0.06	4.6	0.0265	0.013	0.0382
Cassava	*Manihot esculenta* Crantz	18	0.054	5.111	0.0243	0.0105	0.035
Sesame	*Sesamum indicum* (L.)	16	0.048	4.438	0.0189	0.0108	0.0286
Pumpkin	*Cucurbita* (L.)	14	0.042	4.571	0.018	0.0091	0.0255
Cotton	*Gossypium *(L.)	13	0.039	5.846	0.0176	0.0066	0.0253
Sweet potato	*Ipomoea batatas* (L.) Lam	12	0.036	3.083	0.0248	0.0116	0.0278
Eggplant	*Solanum melongena* (L.)	11	0.033	6.091	0.0127	0.0054	0.0198
Fonio	*Digitaria exilis* (Kippist) Stapf	8	0.024	6	0.0099	0.004	0.0145
Tomato	*Solanum lycopersicum* (L.)	6	0.018	6	0.0055	0.003	0.0092
Pepper	*Capsicum annuum* (L.)	6	0.018	5.833	0.0044	0.0031	0.0084
Other		3	0.009	4.333	0.0035	0.0021	0.0041
Melon	*Cucumis melo* (L.)	2	0.006	4.5	0.0036	0.0013	0.0032
Calabash	*Lagenaria siceraria* (Molina) Standl	1	0.003	5	0.0019	6.00E − 04	9.00E − 04
Cucumber	*Cucumis sativus* (L.)	1	0.003	6	0.0013	5.00E − 04	6.00E − 04
B groundnut	*Vigna subterranea* (L.) Verdc	1	0.003	5	6.00E − 04	6.00E − 04	0
Turnip	*Brassica rapa* (L.)	1	0.003	8	4.00E-04	4.00E − 04	0

### Collection and local nomenclature of cowpea varieties

During the survey, 702 cowpea accessions were collected in Thiès (84), Louga (155), Diourbel (158), Fatick (85), Saint-Louis (122), Kédougou (19) and Sédhiou (79) [27]. One to seven accessions were collected per farmer, with an average of two accessions per farmer. These accessions were identified under 59 different local names. The informal interviews with farmers showed that irrespective of their ethnic group, farmers translated “niébé,” the French word for cowpea, into the local language to name the species *Vigna unguiculata var. unguiculata.* In this way, the terms “Niébé” or “Seupe” are used by the Wolof and Halpulaar (Fula and Toucouleur), “Sosso” is used by the Mandinka, “Niao” by the Serer, “Deulleugane” by the Moors and “Oufithion” by the Manjak.

A wide range of reasons is used by the farmers to identify their cowpea varieties. Indeed, 75% of names make reference to morphology (seed color and size or vegetative cycle), 14% are named after a person (the person who brought the variety to the village, a woman’s name if the variety is productive, etc.) and 1% refer to the geographic origin (the zone they came from). Lastly, 9% have names that refer to a specific event (details not provided here) or are arbitrary (Table 6).

**Table 6:** Percentage of name categories for cowpea

Region	Morphology %			Person’s name %	Zone of origin %	Other %	Total %
	Color	Vegetative Cycle	Color/Size				
Thiès	61	5	1	30		3	100
Louga	46	3	6	21	2	22	
Diourbel	73		4	16		7	
Fatick	62		9	22		7	
Sédhiou	96	3		1			
Kédougou	95					5	
Saint-Louis	47	12	4	10	5	22	
% Average	68.57	3.28	3.43	14.28	1	9.43	100

Most of the time, the names of varieties are composed of a generic name for cowpea in the local language plus a second term, which either refers to simple morphological characteristics (seed color), people’s names or zone of origin. Among the Mandinka, for example, cowpea is known by the generic name “Sosso.” In order to identify red cowpea, farmers add the suffix “wouléroung” (red) to the name “Sosso.” In all the regions visited, the names generally referred to morphology, particularly seed color (for example, “niebe bou wekh” or white cowpea). Sometimes seed size is added (for example, “niebe bou wekh bou didji” or white cowpea with large seeds). Some cowpea names are associated with the seeds’ geographic origin (Fouta cowpea) or a person (Baye Ngagne, Mame Fama, Marame Penda). In Senegal, the GOANA agriculture program, launched in 2008 by the former President of the Republic, Abdoulaye Wade, coincided with the introduction of a cowpea variety that is now called after the program. The Goana variety is sometimes called “pea” (because the shape of the seed is quite round or full) or “nenou naat,” which means “guinea fowl’s egg,” in reference to the marks on the seed’s integument (Table 7).

**Table 7:** Local names, English translation and historical references

Local Name	English translation	Meaning
Baye Ngagne	Baye Ngagne or black cowpea	A person’s name
Delleugane Labial	White cowpea	The color of the seed coat
Delleugane Leukhmare	Black cowpea	The color of the seed coat
Fithionouny oufithial	White cowpea	The color of the seed coat
Gouana	Goana	Refer to the agricultural program GOANA
Hectare	Hectare	The seed’s pleasing appearance
Mame Fama	Mame Fama	A person’s name
Marame Penda	Marame Penda	A person’s name
Melakh	Melakh = Flash	The variety early maturing cycle
Mosse kham	Taste to know	The taste
Ndao counda	Ndao counda	A person’s name
Ndiaga aw	Ndiaga aw	A person’s name
Ndiaye wekh	White Ndiaye	The color of the seed coat
Ndieussiw	Ndieussiw	The capacity to produce fodder
Nenou Naat	Guinea fowl’s egg	The color of the seed coat, which has brown speckles
Niao balne	Black cowpea	The color of the seed coat
Niao ndane	White cowpea	The color of the seed coat
Niebe bou wekh	White cowpea	The color of the seed coat
Niebe baledjo	Black cowpea	The color of the seed coat
Niebe bodedjo	Red cowpea	The color of the seed coat
Niebe bodedjo-baledjo	Black-white cowpea	The color of the seed coat
Niebe bou khonk	Red cowpea	The color of the seed coat
Niebe bou khonk bou didji	Red cowpea with big seeds	The seed size and color
Niebe bou khonk bou sew	Red cowpea with small seeds	The seed size and color
Niebe bou nioul	Black cowpea	The color of the seed coat
Niebe bou wekh	White cowpea	The color of the seed coat
Niebe bou wekh bou didj	White cowpea with big seeds	The seed size and color
Niebe bou wekh bou sew	White cowpea with small seeds	The seed size and color
Niebe danedjo	White cowpea	The color of the seed coat
Niebe fouta	Fouta cowpea	Originally from Fouta and mainly used for floodplain cultivation
Niebe Kell	Kell cowpea	
Niebe Koudioule		
Niebe Mame Diarra	Mame Diarra cowpea	A person’s name
Niebe poude	Grayish cowpea	The seed’s faded color
Niebe poury	Grayish cowpea	The seed’s faded color
Oufithion otopeul	Black cowpea	The color of the seed coat
Oufithion oudjankfan	Red cowpea	The color of the seed coat
Pakau	Pakau	
Petit pois	Pea	The seed’s roundish shape
Samba sagnal		A person’s name
Saneba sosso	White cowpea	The color of the seed coat
Seupe bou khonk	Red cowpea	The color of the seed coat
Seupe bou wekh	White cowpea	The color of the seed coat
Sosso fima		
Sosso fing	Black cowpea	The color of the seed coat
Sosso Khoyo		
Sosso koyma	White cowpea	The color of the seed coat
Sosso meunie	White cowpea	The color of the seed coat
Sosso meunie maynama	Late white cowpea	The seed size and color
Sosso missew		
Sosso resse mesengo		
Sosso wouleroung	Pale red cowpea	The color of the seed coat
Tachet	Spotted	The color of the seed coat, which is brown spotted
Tamate awo	First wives’ tomato	The seed’s red color means that less tomato paste is used to prepare rice-based dishes
Walette	Early	The seed’s early maturity
Walette bou nioul	Black Early	The seed’s early maturity and color
Walette bou wekh	White Early	The seed’s early maturity and color
Yacine	Yacine	
Yakhoul tamate	That wastes no tomatoes	The seed’s red color means that less tomato paste is used to prepare dishes

After standardizing the spelling and identifying the synonyms, 36 names of varieties were kept. Irrespective of the ethnic group, the cowpea varieties called white cowpea (26% of all the varieties in the collection), red cowpea (25%) and black cowpea (15%), and Baye Ngagne (9%) are the most commonly grown in Senegal.

**Fig. 5:**
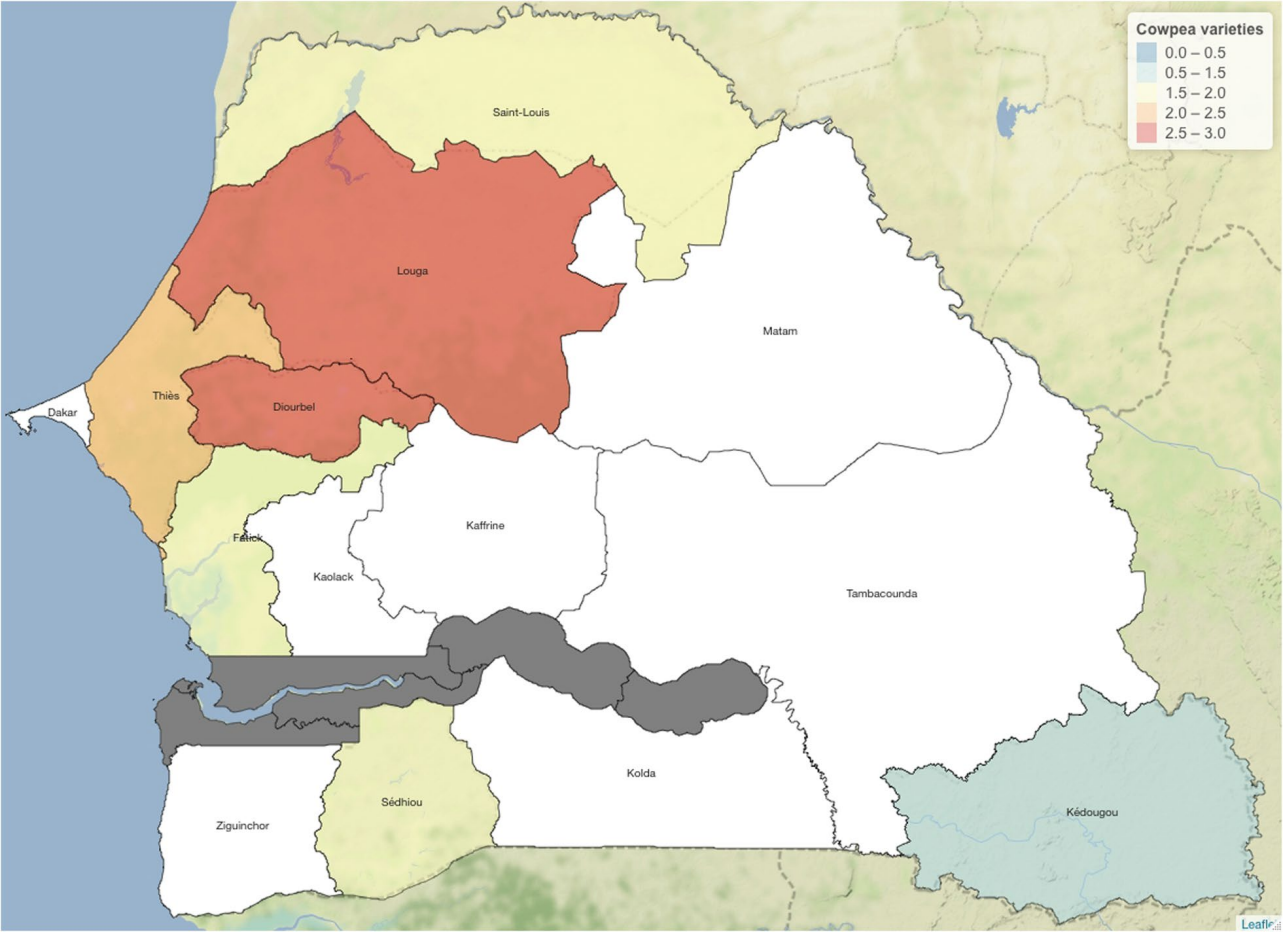
Average number of cowpea varieties by region

The zone in the north and center of the groundnut producing area has the greatest diversity (Louga and Diourbel), whereas Kédougou has the fewest varieties. Cowpea production is more diversified in the regions of Diourbel and Louga, followed by Thiès, Saint-Louis and Sédhiou, respectively (Fig. 5).

The average number of cowpea varieties per farmer ranged from 1 (Kédougou) to 3 (Diourbel and Louga) (Table 8). The Diourbel and Louga regions are also where there is greater linguistic diversity among interviewed farmers. Therefore, the possible link between cowpea diversity and the farmers’ cultural diversity cannot be ruled out.

**Table 8:** Number of varieties per farmer for each region

Regions	No. farmers	No. var/region	No. var/farmer
Diourbel	59	158	2.678
Fatick	48	85	1.771
Kédougou	18	19	1.056
Louga	58	155	2.672
Saint-Louis	62	122	1.968
Sédhiou	52	79	1.519
Thiès	38	84	2.211

### Cropping systems and acquiring seeds

#### Cropping systems

The majority of the farmers interviewed grow cowpea as a single crop (65%). This method of cultivating cowpea is far more frequent in four regions in Senegal, namely Louga, Diourbel, Kédougou and Saint-Louis. Groundnut is the species most commonly associated with cowpea. This association was described for 28% of the farmers surveyed, especially in the regions of Thiès, Fatick, Sédhiou and Diourbel. Cowpea is also associated with maize (3%) in the Saint-Louis region, millet (0.3%) in the Kédougou region and market gardening (0.85%) in the regions of Louga, Saint-Louis and Kédougou (Table 9).

**Table 9:** Cowpea crop associations according to region

Methods	Regions							Total	Percentage%
	Th	Lg	Dl	Fk	Sd	Kg	SL		
Associated with groundnut	42	6	35	49	55	1	9	197	28
Associated with market gardening	0	3	0	0	0	1	2	6	0.85
Associated with maize	0	0	0	0	0	2	20	22	3
Associated with millet	0	0	0	0	0	2	0	2	0.3
Single crop	35	145	117	32	24	13	91	457	65
Single crop associated with groundnut	7	1	6	4	0	0	0	18	2.6
Total	84	155	158	85	79	19	122	702	100

*Th* Thiès; *Lg* Louga; *Dl* Diourbel; *Fk* Fatick; *Sd* Sédhiou; *Kg* Kédougou; *SL* Saint-Louis

In the regions of Thiès, Fatick, Diourbel, Kédougou and Sédhiou, cowpea is grown in the rainy season. In general, sowing is in June and July (53.42%) and harvesting is in September and October (93.44%). In the Louga region and part of the Saint-Louis and Diourbel regions, sowing is in August and September (42.60%) and harvesting is in November. Floodplain cultivation of cowpea is only found in the Saint-Louis region (3.99%). For this type of production, sowing occurs between November and January and harvesting is between February and March.

There are three groups of cowpea varieties grown in Senegal that can be distinguished according to their development cycle: early (number of days < 70), semi-early (between 70 and 90 days) or late (number of days ≥ 90). The early maturity varieties represent 81.34% of the varieties grown. They are found in all regions, except Kédougou. Semi-early varieties (3.84%) are grown in Louga and Diourbel. Lastly, late maturity varieties (14.67%) are generally grown in the regions of Kédougou, Thiès and Saint-Louis (Table 10).

**Table 10:** Cropping calendar and cycle of accessions in each region

Features	Conditions	Regions							Total	%	Chi-Square		
		Th	Lg	Dl	Fk	Sd	Kg	SL			Value	df	*p*-Value
Sowing date	June–July	83	34	103	81	63	11	0	375	53.42	455.5^a^	12	< 0.001
	Aug–Sept	1	121	55	4	16	8	94	299	42.60			
	Nov–Jan	0	0	0	0	0	0	28	28	3.99			
Total		84	155	158	85	79	19	122	702	100.00			
Harvest date	Sept–Oct	84	155	158	84	79	10	86	656	93.44	302.6^a^	18	< 0.001
	Nov	0	0	0	0	0	8	8	16	2.28			
	Feb–March	0	0	0	0	0	1	28	29	4.13			
	DNR	0	0	0	1	0	0	0	1	0.14			
Total		84	155	158	85	79	19	122	702	100.00			
Cycle	< 70	45	133	148	81	79	3	82	571	81.34	251.955^a^	18	< 0.001
	70–90	0	16	7	2	0	1	1	27	3.84			
	≥ 90	39	6	3	1	0	15	39	103	14.67			
	DNR	0	0	0	1	0	0	0	1	0.14			
Total		84	155	158	85	79	19	122	702	100.00			

The *p*. value of the chi^2^ test for the sowing dates, harvest dates and length of cycle is below 0.001. The hypothesis of the independence between these variables and the regions has been rejected as a result. *Th* Thiès; *Lg* Louga; *Dl* Diourbel; *Fk* Fatick; *Sd* Sédhiou; *Kg* Kédougou; *SL* Saint-Louis; *MS* sowing date; *MR* harvest date; *June*-*July* June and July; *Aug*-*Sept* August and September; *Nov*-*Jan* November to January; *Sept*-*Oct* September and October; *Feb*-*March* February and March

#### How seeds are acquired

Most of the interviewees (57%) stated that they obtained their first cowpea seeds at markets or from seed suppliers, NGOs, cooperatives or farmer organizations outside the village. Forty-two percent (42%) obtained them from relatives or neighbors in the village. How seeds are acquired varies depending on the region (Table 11). Eighty-one percent (81%) of interviewees stated that they acquired their first seeds in the last two decades, compared to only 11%, who obtained their seeds more than 25 years ago. More than 6% of interviewees cannot remember the year when they acquired their seeds. The majority (68%) of seeds from the last season were home-grown (Table 11).

**Table 11:** Origin of the seeds grown by the farmers

Nature	Conditions	Regions							Total	%
		Th	Lg	Dl	Fk	Sd	Kg	SL		
Place where first acquired	Outside the village	43	95	68	60	48	0	85	399	57
	Village	41	57	86	25	31	18	37	295	42
	DNR	0	0	0	0	0	1	0	1	1
Total		84	155	158	85	79	19	122	702	100
Year when first acquired	˂25	66	122	106	73	79	13	115	574	81
	˃25	4	13	42	9	0	6	6	80	11.4
	DNR	14	20	10	3	0	0	0	47	6.7
Total		84	155	158	85	79	19	122	702	100
Home-produced	N	27	52	35	21	16	5	62	218	31
	O	57	103	123	64	63	14	59	483	68
	DNR	0	0	0	0	0	0	1	1	0.14
Total		84	155	158	85	79	19	122	702	100

*Th* Thiès; *Lg* Louga; *Dl* Diourbel; *Fk* Fatick; *Sd* Sédhiou; *Kg* Kédougou; *SL* Saint-Louis

## Discussion

Drawing on the new collections and the recent surveys, which were more exhaustive than earlier surveys, the aim of this study was to characterize the farming practices associated with growing cowpea in Senegal. It focused particularly on the range of species grown in association with cowpea. The richness and variability of cowpea varieties were established in reference to the farmers’ nomenclature. We also identified where farmers obtained their seeds.

### Diversity of species grown with cowpea

In all the zones surveyed, cowpea producers also grow groundnut and millet. In Senegalese farming systems, these three species are complementary. Along with sorghum, cassava, watermelon and red sorrel, they are the main cash crops grown in the center and north of the groundnut growing area, which is ideal for growing cowpea. Our findings on the regional distribution of species diversity are similar to those obtained when the FAO conducted inventories of the agricultural species in rural areas [28], in which Eastern Senegal and the Casamance appeared to be priority areas for plant breeding resources and crop biodiversity. This can be explained by the abundant rain in these zones, the diversified traditional farming practices, the ethnic diversity and, lastly, the proximity of the region to neighboring countries, which favors exchanges. Although the Sédhiou region has as much rain as South-East Senegal (Kédougou), it has less species diversity. The Saint-Louis region is still diversified in terms of cultivated species, despite its rainfall deficit. This region’s geographical position offers favorable climatic conditions for farming. The potential in terms of irrigable land, estimated at 172 800 ha, and the abundance of water [29] no doubt contribute to this diversity as well.

### Cropping system

The majority of farmers surveyed grow cowpea as a single crop. This cropping system is found in the regions of Louga, Diourbel and Kédougou. In the groundnut growing area, which includes the regions of Diourbel and Louga, there has been a rainfall deficit for decades. However, cowpea is adapted to these conditions. More and more land is being used to grow cowpea. Between 2012–2017, cowpea was grown on 165 452 ha, on average. This increased to 257 219 ha in 2019 [30]. In these zones, where the harvest is destined for sale, cowpea is grown in huge fields. In contrast, in other regions, cowpea is considered as a subsistence crop and is associated with other crops, such as groundnut, maize, millet or even market gardening. Polyculture is practiced by farmers who do not have large areas of cultivable land. This association with other crops is used as a strategy to reduce the risks of production loss due to climatic hazards.

In the regions of Thiès and Louga, young people grow cowpea, which could help reduce immigration. In fact, in this part of the country, the legume is grown as a cash crop on large areas of land. In the Sédhiou region, young people also grow cowpea, although it is often neglected in favor of other crops. This could be explained by the fact that varieties from other crops are better adapted to the groundnut producing zone, such as the Sédhiou region. In Sédhiou, cowpea is traditionally valorized by women. In the regions of Diourbel, Fatick and Saint-Louis, cowpea is grown by aged farmers, who probably know more about traditional accessions and their cropping practices.

### Cowpea’s area of distribution and varietal richness

This study helped to confirm the area of distribution of cowpea production in Senegal. In fact, in the regions of Diourbel, Louga, Thiès and Saint-Louis, collecting several varieties from one farmer is common, whereas in the Sédhiou and Kédougou regions, cowpea is less common and, on average, there is seldom more than one variety per farmer. The cultivation of this legume is more diversified in Diourbel and Louga. This reveals the importance and richness of the species in the central north and north, the main cowpea growing areas in Senegal [31]. The department of Louga, which is in the center of this region, appears to be the preferred zone for growing cowpea: 21% of cultivated land is used to grow this species [32].

The analysis of diversity based on the local names for cowpea allowed us to identify six appellations for the cowpea species. On a varietal level, 59 different names were identified. Varieties whose seeds have the same morphological features may have different names depending on the ethnic group. These names essentially refer to seed color, size or people’s names. Thus, the farmer manages diversity by recognizing perceptible characteristics, especially morphological features [33]. By studying the classification processes, we were able to determine the biological diversity of cowpea, as perceived by farmers. The diversity of the local names is an indicator of the plant’s importance in a geographic environment [34]. In Senegal, the fact that the local names that designate cowpea vary depending on locality or ethnic group was reported a long time ago [35]. This observation suggests that there is a close link between farmers’ cultural diversity and varietal diversity. A high level of diversity was also mentioned for fonio, with 52 local names [36], and maize, with 81 local names [37]. In this study, seed color is the most distinctive element and the most often used by farmers for naming varieties. This naming process can cause confusion between traditional and improved varieties because the latter’s names are sometimes constructed in the same way. For example, the improved variety, Yacine, is called “Niebe bou khonk” in Wolof, which means “red cowpea.” In Burkina Faso, names are constructed using eye color (in over 35% of cases) and seed size (almost 45%) [38]. According to Ouedraogo et al., color and texture are only used for less than 10%. However, our findings, which are in line with the studies by Dabat et al. [39] in Burkina Faso, show that white varieties appear to be more valued because the majority of seeds used by farmers are white.

Cowpea is mainly grown during the rainy season in all the zones surveyed, except the Saint-Louis region, where cowpea is also grown on the floodplain. Three groups (early, intermediary and late) were identified according to the varieties’ development cycle. According to Kouakou et al*.* (2007), on a local level, cowpea diversity is generally due to its phenological adaptability to environmental constraints. The abundance of early maturing accessions may be due to the adoption of improved varieties that are early. Late varieties are no longer grown in the main cowpea producing areas because rainfall has been irregular or insufficient for four decades. This may also explain the high number of early varieties. The earliest varieties were collected in Sédhiou, which has the longest rainy season. However, in this region, very small areas were cultivated for home-consumption. The variability of rainfall in the different regions could explain the phenological diversity observed. In fact, more late accessions are grown in the Kédougou region, where rainfall is higher, and in the Saint-Louis region, where floodplain cropping plays an important role. These types of varieties are valuable because they are dual purpose and can be used as seed and fodder. In fact, under favorable conditions, they produce a large amount of seeds and fodder [3]. The late varieties found in the regions of Thiès, Kédougou and Saint-Louis could constitute an important pool for local and traditional varieties. In the cereal growing region of Thiès (where 47.2% of land is cultivated with maturing cowpea) [40], late maturing cowpea has a positive effect on cereal yields in the crop rotation because it produces huge quantities of biomass [41].

### The seed supply

In the last two decades, most of the seeds in the farmers’ possession were purchased at the market or obtained from agricultural services, NGOs, farmers’ organizations and cooperatives. These types of structure are common to several villages. Consequently, the same variety can be found in different villages or regions, even if it has different names. Thus, the pleasing appearance of seeds of one cowpea variety can encourage people to buy it at a market, even if they are unaware of its germination performance and agricultural value.

Many of the people surveyed obtained their first seeds in the village, either through donations or by trading with relatives, friends or neighbors. Similarly, married women obtain their first seeds from their husband or parents-in-law, along with plots of land, after leaving their place of birth to go to their husband’s place of residence. Thus, women rarely take seeds from their home or continue to obtain seeds from their relatives, especially if they live in different villages.

The majority of seeds from the season preceding this study were home-grown. In fact, farmers keep a share of their previous harvest for seed. Consequently, farmers only purchase or obtain seeds at the market or from relatives or neighbors the year after a poor harvest or a food shortage.

## Conclusion

Identifying the nomenclature for the local cowpea varieties and their seed management system is essential for optimizing local diversity. This study revealed the considerable diversity of local names. This diversity is an indicator of the importance of cowpea in Senegalese farming systems. The names primarily refer to the seed morphology or color, a feature that facilitates identification. The named diversity of cowpea is greater in regions where the crop systems are less diversified. In the studied area, more than half the cowpea seeds grown by farmers are obtained from markets, NGOs, agricultural services and projects and then farmers produce and conserve their own seeds. Cowpea is generally grown as a single crop or associated with groundnut or maize. The length of the growing cycle is rarely used by farmers to identify their varieties. However, we classified varieties in terms of development cycles because of the difference observed between sowing and harvesting dates. This study made it possible to characterize the diversity of cowpea grown in Senegal. Undoubtedly, the diversity of farming practices and cowpea cropping systems is closely linked to the diversity of the biological types grown in the country and vice versa.

## Supplementary Information

**Additional file 1**: Collection areas and socio-cultural characteristics of surveyed farmers.

**Additional file 2**: The various species cultivated with cowpea in the areas visited.

**Additional file 3**: List of the 702 cowpeas accessions, their local names and signification, regions of collection, acquisition and cycle.

## Data Availability

The data supporting the results are presented in the tables of the article. More details can be requested of the corresponding author.
